# The Influence of Parents, Coaches, and Peers in the Long-Term Development of Highly Skilled and Less Skilled Volleyball Players

**DOI:** 10.3389/fpsyg.2021.667542

**Published:** 2021-04-30

**Authors:** Patrícia Coutinho, João Ribeiro, Sara Mesquita da Silva, António M. Fonseca, Isabel Mesquita

**Affiliations:** Centre for Research, Education, Innovation and Intervention in Sport (CIFI2D), Faculty of Sport, University of Porto, Porto, Portugal

**Keywords:** social influences, talent development, athlete development, expertise level, volleyball

## Abstract

The purpose of this study was to understand the perceptions of highly skilled and less skilled volleyball players about the influences that parents, coaches, and peers had on their sport development and performance achievement. Highly skilled (*n* = 30) and less skilled (*n* = 30) volleyball players participated in semi-structured retrospective interviews to explain how parents, coaches and peers may have influenced their sport participation. Data was analyzed through a process of content analysis. Results indicated that parents, coaches, and peers had an important influence in player's sport development but differing according to players' expertise level. Concerning to parental influences, tangible support during the early years of development was mentioned by all players. However, parents' level of involvement and parenting styles revealed interesting differences between highly skilled and less skilled players. Highly skilled players perceived a moderate parental involvement and an autonomy-supportive parenting style, while less skilled players referred a excessive parental involvement in players' sport participation. Coaches influences showed to have some similarities in the early years with all players mentioning coaches as caring and recognizing their value as an athlete. However, highly skilled players described a different training environment characterized by a demanding coach, individualized instruction, and specific goal setting. Regarding peers' influence, all players recognized that friends were not only one of the main reasons to start playing volleyball, but also an important source of support to remain engaged and staying motivated to do sport. Highly skilled players, however, mentioned the importance of teammates' positive push and critiques during practice for enhancing their motivation, team cohesion and friendship. They also highlighted the importance of friends outside of sport in the later years of their career by acting as an escape from all the pressure that emerged from volleyball training and competition demands. Overall, these findings highlight different social influences according to the players' expertise level suggesting the need to examine more extensively the nature of significant others' support on athlete and talent development.

## Introduction

Sport is a privileged social context that provides rich opportunities for interpersonal interaction. The social network established throughout athlete's sport participation, in combination with the fairly intensive nature of this involvement, plays an important role in how they experience sport. Particularly, the social agents that directly interact in those experiences such as parents, peers and coaches, may have significant implications in athletes' sport participation, personal development, and performance (Côté, 2002; Côté and Hay, 2002; Côté et al., 2007, 2012).

Parents play a particularly important role during the early years of athlete development (sampling years) by giving the opportunity for their child to experience several sports and providing the resources needed to nurture their sport development (Côté et al., 2007; Harwood et al., 2012; Knight and Holt, 2014; Knight, 2016). As the athlete progresses in sport, the parents' role changes from a leadership role during the sampling years to a follower and supporter role during the specializing and investment years, where the facilitation of an optimal environment for athletes' development is the major concern (Côté and Hay, 2002; Côté et al., 2007; Harwood et al., 2012; Knight, 2016; Knight et al., 2016). Here, autonomy-supportive parents (i.e., parents who support their child's sport involvement and allow them to have an active role on their decisions) have been associated as a positive influence on children's sport development (Lauer et al., 2010b; Knight et al., 2011; Barreiros et al., 2013; Fraser-Thomas et al., 2013; Keegan et al., 2014; Knight and Holt, 2014). On the contrary, parents' over involvement in children's sport participation as well as the supply of an excessive sport-related feedback can act as sources of pressure, which may consequently decrease motivation and enjoyment in sport (Gould et al., 2008; Lauer et al., 2010a,b; Harwood et al., 2012).

Besides parental influences, the coach has a critical role in optimizing athletes' development through sport (Côté et al., 2010; Chan et al., 2012; Cushion et al., 2012; Atkins et al., 2015; Mesquita et al., 2015). Literature has suggested that during the sampling years the role of the coach is supportive, caring and encouraging (coach as a sport-helper and child-centered), while in the specializing and investment years coaches are described as more qualified, knowledgeable (sport specialist), and performance oriented (Potrac et al., 2002; Côté et al., 2007; Fraser-Thomas et al., 2008b; Barreiros et al., 2013; Jones et al., 2013). Coaches are also responsible for designing practices, encouraging their athletes to train on a long-term basis, and giving instruction (Barreiros et al., 2013). For example, time devoted for individual instructions with athletes usually differentiates not only those who prolong their engagement in sport from those who drop out (Fraser-Thomas et al., 2008a,b; Fraser-Thomas and Côté, 2009), but also experts from non-experts (Baker et al., 2003; Barreiros et al., 2013). Studies have also shown that coach behaviors have a significant impact on athletes' affective responses. For example, coach reinforcement, encouragement, individualized instruction, guided goal setting, and a belief in athletes' capabilities are associated with athletes' positive experiences in sport such as increased motivation and enjoyment in sport (Fraser-Thomas and Côté, 2009; Cushion et al., 2012; Barreiros et al., 2013; Mesquita et al., 2015).

Finally, peers can also play an important role throughout athlete development. During the sampling years, peers are one of the main reasons why children participate in organized and unorganized sports, as well as remain involved and motivated to practice sports later in their development (Côté et al., 2007; Keegan et al., 2010; Chan et al., 2012; Barreiros et al., 2013). As the athlete progresses in sport (up to the investment years), friends outside of sport are considered an important source of support since they tend to fulfill athletes' motivational and emotional needs (Côté et al., 2007; Fraser-Thomas et al., 2008a,b; Barreiros et al., 2013; Bruner et al., 2013). Studies that examined the association between peer relationships and athletes' affective outcomes have suggested that positive reinforcement and the motivational atmosphere provided by peers during practice are positively linked to athletes' motivation toward sport (Vazou et al., 2005; Keegan et al., 2010, 2014; Smith et al., 2010). Despite the importance of these social agents in athlete's sport participation, comparatively little attention has been given to the specific role of peers on athlete and talent development.

This body of research has been supported in the literature as contributing to a better understanding of the role of social influences in athlete and talent development. Notwithstanding, most studies have provided an isolated perspective of those influences, examining the influence of parents, coach and peers in an independent way. Future studies would therefore benefit from an ecological rational that examine all these social influences in an integrated viewpoint. Here, a more contextual approach will provide a better understanding of the interrelatedness between the athlete and the context contributing with richer evidence about the influence of the social environment on athlete and talent development. Moreover, this research topic is in a clear need of sport-specific studies to better understand the idiosyncratic behaviors of parents, coaches, and peers based on the sport itself and the developmental stage of the athlete (Lauer et al., 2010a,b; Atkins et al., 2015; Harwood et al., 2019). For instance, the unequal developmental pathways taken by athletes from sports where peak performance occurs before or after an athlete has full matured may imply different patterns of parents, coaches, and peers support throughout athlete development. Particularly in volleyball (where peak performance is achieved during adulthood) (Balyi and Hamilton, 2004; Coutinho et al., 2016), no studies have attempted to specifically understand the influences of parents, coaches and peers throughout the players' development.

Therefore, the purpose of this study was to understand the perceptions of highly skilled and less skilled volleyball players about the influences that parents, coaches, and peers had on their long-term sport development and performance achievement.

## Materials and Methods

### Philosophical Perspectives and Design

A qualitative descriptive approach was considered in this study. Qualitative descriptive studies are particularly useful when exploring applied topics and used to understand the “who, what, and where of events or experiences” (Sandelowski, 2000, p.338). Considering that this study was interested in understanding the behaviors and influences of parents, coaches, and peers throughout the volleyball players' long-term development, a qualitative descriptive approach was deemed appropriate. This study was positioned within the interpretivism paradigm, underpinned by ontological relativism and epistemological constructionism. That is, within this study, it was assumed that reality is multiple and subjective and that knowledge is socially constructed (Weed, 2009). The rational for this approach was to understand the players' experiences with parents, coaches, and peers in sport, whilst acknowledging that their views are reflective of their own sporting contexts. This study focused on understanding the influences of social agents (parents, coaches, and peers) in the development of volleyball players from different expertise level and gender. The focus on different players (i.e., highly skilled/less skilled, male/female) was driven by a recognition that the participants' lived experiences may differ according to their cultural, social, and personal context. As such, the players analyzed in this study was selected based on two main criteria: (1) expertise level (considering their competitive level and previous sporting experiences; defined as highly skilled and less skilled players), and (2) gender (male and female players). The aim of such criteria was to achieve maximal variance between participants. The heterogeneity of participants, the diversity of contexts and the variety of lived experiences are factors of particular importance to understand the phenomena under study.

### Participants

Sport Coaching staff members of 18 volleyball clubs in Portugal were recruited to help select participants for the study. Participants included 30 highly skilled and 30 less skilled volleyball players (15 male and 15 female for each group). Taking into account the philosophical perspective and design of this study, a panoply of factors is related with the topic of this study (i.e., social influences in sport). For that reason, only with a diversified sample characterized by its interindividual variety (15 highly skilled male, 15 highly skilled female, 15 less skilled male, 15 less skilled female), we were able to capture a complete picture of the influence of parents, coaches, and peers in the development of volleyball players. The average age of highly skilled players was 29.6 years (*SD* = 3.4) and the average age of less skilled players was 28.9 years (*SD* = 3.1). Participants were selected based on two main criteria: (a) being no younger than 23 years old (peak performance in volleyball is achieved in the mid to late twenties) (Balyi and Hamilton, 2004), and (b), having a minimum of 10-years of sport-specific experience in volleyball, but with no prior limitations on the number of reported hours spent in sport participation. Additional criteria that we used to characterize the sample of highly skilled participants included: belonging to a senior national team (Memmert et al., 2010; Hayman et al., 2011) and being ranked amongst the best volleyball players by national team coaches (Berry et al., 2008). The less skilled players were regularly involved in organized competitive volleyball but competing in a lower level (third league) and had never been part of a senior national team.

All procedures followed the guidelines stated in the Declaration of Helsinki and were approved by the ethics committee of the first author's institution. Players were contacted personally or by telephone and were provided with an overview of the study–100% of the players contacted agreed to participate in the study. Prior to the beginning of the study, all players were given information sheets that informed them about the purpose of the study and signed consent forms.

### Procedure and Data Collection

Semi-structured interviews were deemed the most appropriate method of data collection for this study because they allow in-depth information to be gained from participants discussing their interpretation of the influence of significant others (i.e., parents, coaches, and peers) in their sport development (Cohen et al., 2001; Denzin and Lincoln, 2005). This was particularly important in the study considering the subjective and individual nature of the topics under study.

A semi-structured interview guide was developed based on the retrospective interview procedure suggested by Côté et al. (2005). The interview design sought to gain an in-depth understanding of the influence of significant others (i.e., parents, coaches, and peers) in players' sport development.

The interview began with a statement of what was being studied and a brief explanation of the concepts under study (i.e., types of parents, coaches and peers behaviors, influences and support within the sport context). Following this, participants provided their names and ages for the tape and briefly explained their pathway in volleyball. This process facilitated subsequent questions and transcription—it also acted to “break the ice.” After this initial phase, the interview continued with questions intended to assess the influences (positive and negative) of parents, coaches and peers on player's sport development. These included: parents' tangible support, parents' emotional support, coaches behaviors and leadership style, coaches' emotional support, peers behaviors, and peers' emotional support. Probing and follow-up questions were used to encourage athletes to expand their answers and also to allow the participant to think in a different manner, such as “Can you give me a specific example of your parents behaviors during that time?”

All interviews were conducted by a female interviewer, who, apart from having experience in qualitative research methods, was also a qualified coach with a high level of volleyball playing experience. This served to facilitate the development of rapport with the participants and a closeness to the phenomena under investigation. Prior to carrying out the interviews, two pilot interviews were completed with participants. These lasted ~60 min and were taped and reviewed with the co-authors. Pilot interviews highly contributed to increase the research quality of this study since allowed the identification of the need to modify questions or other procedures that do not elicit appropriate responses or enable the researcher to obtain rich data (Malmqvist et al., 2019). This allowed the interviewer to rehearse and refine the interview procedure, including the intelligibility of the questions, an improvement in the clarity of the questions, the use of more accessible language for the participants, and the efficient use of elaboration and clarification probes (Miles and Huberman, 1994; Johnson, 1997).

Before each interview the participants were provided with a written and verbal introduction. The introduction outlined the research, reassured confidentiality and included definitions of the main research concepts. Participants were reminded that they were free to discuss any issues they felt were relevant to the topic. Informed consent was obtained and any questions were answered. Interviews were conducted in a quiet and free of distraction location by the primary researcher. The interview was conducted in a one-on-one format and took ~1–2 h to complete. All interviews were audio recorded and transcribed verbatim.

### Data Analysis

All interviews were digitally recorded, transcribed verbatim and checked for accuracy by the research team. Pseudonyms were assigned to each participant to ensure confidentiality throughout the analysis process. Content analysis (Sparkes and Smith, 2014) was used to analyzed the data. The initial stage involved immersion and familiarization with the transcribed data. Specifically, this involved reading the interviews texts several times and identifying segments of data containing meaningful information. The second phase involved the production of initial codes from the data, and basic segments deemed meaningful, were attached labels. This process developed with pre-existing research aims in mind (deductive), alongside openness to new segments (inductive), and was completed manually by-hand. The third phase involved the creation of themes by addressing concepts, and sorting codes into themes. Constant comparison (Weed, 2009) was employed, leading to the amendment of themes for the initial phase four grouping of overarching themes, themes and subthemes. Once themes were reviewed and defined, the last phase involved going back through the data to name the identified themes in a more representative demise.

### Methodological Rigor

A number of steps were introduced to enhance the trustworthiness of the findings as well as their credibility and dependability (Johnson, 1997). Firstly, engaging in pilot interviews (and maintaining the same interviewer throughout) helped to maximize interview consistency. The experience of the interviewer as a player and coach also encouraged an affinity with participants. The interviewer previously reflected on the themes under study considering her experiences as a player and coach and awareness was taken before starting the interviews in order to guarantee an impartial position. Secondly, all interview transcripts and a summary of the results were returned to participants for member checking and participant feedback (Johnson, 1997; Cohen et al., 2001). Participants were asked to review their transcripts for verification, which allow them the opportunity to add, delete, or rework any data that they felt did not accurately reflect their intended communications (Miles and Huberman, 1994). After that, participants were contacted by the lead researcher via telephone to undertake a short discussion about the nature of the findings. This process enabled the lead researcher to pose questions to verify that the participants had indeed discussed the topic appropriately. All participants confirmed their transcripts. Finally, two members of the research team were involved in a collaborative approach within the interpretational analysis, with regular meetings to discuss the emerging categorical organization system. These meetings and reflections challenged the lead researcher's decisions and constructions of knowledge, developed interpretations, and offered alternative explanations for the findings. Following an in-depth discussion, the themes that remain doubts or disagreement were re-analyzed and reworded to more accurately portray the data. Through this discussion and questioning the final coding scheme was agreed upon. This important process contributed to the trustworthiness of the data, ensuring the interpretative validity while minimizing the risk of individual research bias (Silverman and Marvasti, 2008).

## Results

Through the analyses of the data, it became apparent that parents, coaches and peers had an important influence in player's sport development but differing according to players' expertise level. In the following sections, types of parent, coach and peer involvement and behaviors that influence the players' development are presented. Results are organized showing the commonalities and differences in these behaviors considering the player's expertise level. Corresponding quotes are provided to illustrate and clarify each theme.

### Parental Influences

#### Highly Skilled and Less Skilled Players: Parents as Providers of Sport Experience

Both highly skilled and less skilled players described and explained how their parents provide a variety of opportunities to practice different sports throughout childhood and how this was important for them as an athlete to choose the right sport. They also referred the importance of parents provided them the resources, equipment, and all the financial and logistic support needed for their sport participation.

“*My parents were tireless during my sport involvement. They provide me the opportunity to try different sports and gave me all the equipment I needed. They were always concerned that I had enough food and sleep… Now I recognize they provided me with the best conditions possible so I was calm and able to practice and compete…” (Highly skilled male #13)*.

#### Less Skilled Players: Parents' Excessive Involvement in Players' Competitive Sport

The most commonly reported description of less skilled players' parents was how they were overly involved in their competitive sport participation, being present in every event of their sport experience, such as competitions, informal tournaments, friendly matches, and training sessions. They mentioned this involvement became a way of pressure for them. They also acknowledged this excessive involvement was a negative influence for their sport development and performance.

“*My mom was always there… not only in my official games, but also in the majority of my training sessions. Sometimes was tough to see her always there because it seemed like she was judging me…” (Less skilled female #3)*.

#### Highly Skilled Players: Parents With a Moderate Level of Involvement in Player's Competitive Sport and Autonomy-Supportive Parenting Styles

Highly skilled players outlined their parents had a moderate involvement in their competitive sport participation (volleyball). This was characterized by being present in important moments for the player such as official competition like finals, but not present, for example, in trainings or informal competitive events. Highly skilled players explained how this behavior has benefitted their sport development. They also mentioned how their parents provided supportive feedback and how this was important for their motivation and commitment. Highly skilled players also highlighted that parents always gave them autonomy to decide which is the best path in their sport participation. They explained that when it comes to decide which club they will play in the next year or to negotiate their contract, for example, parents were there to listen to them and provide their opinion but did not meddle in their decisions.

“*My parents have never been too much involved… they only came to watch my games when I asked them. However, they were always very careful and sought to know how I was doing… It seems like they were not physically there, but they were always in the background…” (Highly skilled male #6)*.

“*My parents have never opposed to anything about volleyball. All they want is to see me happy playing volleyball. They always encouraged me to do my best and always supported through good and bad times…” (Highly skilled female #14)*.

“*When I had to decide something about my career, they never intruded and always let me decide. For example, when I had to move to a different club I always asked them their opinion. They were always there for me and to listen to me. They provided their opinion but never said something that interfere in my decision. In the end, the decision was mine and I decided what was the best for me…” (Highly skilled female #14)*.

### Coach Influences

#### Highly Skilled and Less Skilled Players: Coach as Caring in the Early Years

Both highly skilled and less skilled players explained how their coaches in the early years of development were friendly, affectionate, and passionate about volleyball. These coaches' characteristics were very important to motivate them to start practicing volleyball and to stay involved and committed to the sport. They highlighted the importance of their first coach for their sport development and learning about the sport.

“*I liked my first volleyball coach so much! She was so sweet and managed to motivate me a lot in that time. She loved volleyball and I think that's why I started to love volleyball too. She was always worried about us, about our school… she was like a second mother for me…” (highly skilled female #5)*.

#### Highly Skilled and Less Skilled Players: Coach Recognition of Player's Value

Both highly skilled and less skilled players described the importance of having their value acknowledged by coaches throughout their development. They reinforced the importance of coaches belief in their potential as an athlete and always helping them to evolve in volleyball by providing feedback, reinforcement, attention, and specific exercises. They also stated that coaches gave them the opportunity to exhibit their skills and encouraged them to be better than they were.

*It was important for me to feel that they (coaches) were interested in me and I was not only one more player…they believe in me and in my development…they never ignore me and always believe that I would achieve some degree of success (Less skilled male #10)*.

#### Highly Skilled Players: Demanding Coach

Highly skilled players mentioned how their coaches were tough, rigorous, and demanding during adolescence and how this was important for their expertise achievement. These behaviors, and specially coaches' demand, were represented in the way they want the things to be done. The exercises, the technical actions, the tactical positions, the understanding of the game, the overall commitment, they always tried to get the best out of the player. Their coaches were very committed in providing high level training practices, attention to technical and tactical development and push them to the limit so as they were able to achieve a high level of performance whenever possible. This demanding training environment was perceived by highly skilled players as beneficial for their development and they never considered it as excessive, negative or prejudicial for them.

“*The majority of my coaches always gave me a lot of attention and push me to the limit during adolescence …they were very tough with me, providing lots of attention to technical and tactical details and I knew that's because they wanted me to do my best and achieve a high-level standard.” (highly skilled female #15)*.

“*Trainings were very demanding. We were there to do our best. Or even more, if possible! However, I never felt it was too much. I never felt my coaches passed the limit. I knew that they needed to be like that to get the best out of us. Actually, I always like that type of training environment because I knew I will improve my performance…” (highly skilled male #8)*.

#### Highly Skilled Players: Individualized Coaching and Goal Setting

Highly skilled players referred to having coaches with a very specialized knowledge and experience in volleyball, which was determinant for having high quality training practices. They also mentioned their coaches provided them one-on-one instruction and established specific targets particularly in later stages of development. They considered this individual consideration was especially important for their development because it guided them to know what they need to work and focus on. They also acknowledged that all these behaviors were determinant for achieving high levels of performance later in their careers.

“*They knew very well what they were doing. The targets of the team were well-established and common to everyone, but they also set very specific goals for each player… This was important because we knew what we had to do and what we needed to improve. Because of this, they spent a lot of hours with me after the training session, working on my difficulties… their feedback used to be highly specific and I improved my performance because of that.” (Highly skilled male #5)*

### Peers Influences

#### Highly Skilled and Less Skilled Players: Friends as a Reason to Play Volleyball

Both highly skilled and less skilled players referred that friends were the reason to start playing volleyball and remain involved in this sport. Friends were already practicing volleyball when they started and were important for their integration in the sport and the team/group. This positive first experience helped them to enjoy practicing volleyball at that time and stay involved in this sport throughout the time.

“*I start playing volleyball because all my friends played at that time… Then it was easy to start enjoying that sport…” (Less skilled male #3)*.

“*I remained involved in volleyball in the first years definitely because of my friends/teammates…” (Highly skilled female #8)*.

#### Highly Skilled and Less Skilled Players: Friends Outside of Sport as a Positive Influence

Both highly skilled and less skilled players described friends outside of sport (i.e., friends that were not involved in volleyball) as an important source of support and encouragement to play volleyball. They mentioned the importance of their friendship and invitation to social events despite their busy agenda related to volleyball (training sessions and competitions). They highlighted how positive is this understanding about their sport career and recognized the contribution of these behaviors to a stable psychological state and well-being, which was favorable for their development as an athlete.

“*It was very important to have their friendship. Although I was very busy with volleyball, they still invited me to parties and social programs and never forgot me! This was very important for me and all this was then reflected in training and competition, because I was feeling good with myself and feeling that everyone supported me…” (Highly skilled male #13)*.

#### Highly Skilled Players: Teammates Positive Push

Highly skilled players indicated that teammates provided a stimulating motivational climate during training practices. They explained that teammates' push and critiques during practice were important for training with higher quality. Teammates' push and critiques are represented in behaviors such as asked the player to do more and better when he/she were not doing, providing corrective feedback concerning technical and tactical actions, establishing rivalry and a competitive environment when they are playing. They also mentioned this motivational climate promoted by teammates was positive since enhanced team cohesion, friendship and players' motivation to achieve better results. They never felt this motivational climate was negative for them. On the contrary, they believed this training environment contributed to potentiate their development and the achievement of high levels of performance.

“*The competitiveness between us was an important ingredient for our success…this was an important factor in enhancing our motivation and making us work more and more… At the end of the training we were all friends again, but in the practice we were like warriors!” (Highly skilled female #4)*.

“*They [teammates] were very important for me. They were always there to push me when I am not totally committed to do my best. They helped me to know what I was doing wrong technically or tactically and corrected me. Also, when we were doing six vs. six [playing/preparing the game] they were very competitive and the rivalry present in the game was amazing! I never felt that was a bad thing… I know that only with that kind of thing I would achieved my best! (Highly skilled male #12)*.

#### Highly Skilled Players: Friends Outside of Sport as an Escape in the Later Years

Highly skilled players described how their friends outside of sport acted as an escape from all the pressure and stress of volleyball in the later years of their career. They mentioned they were important to think and talk about other issues than volleyball and therefore relax from all the pressure and negative things related to their sport career.

“*I really try to have other things besides volleyball… There is a lot of pressure to win so my friends are very important because when I go out with them I know that I will talk and think about other things rather than volleyball…” (highly skilled male #7)*.

## Discussion

The present study sought to understand the perceptions of highly skilled and less skilled volleyball players about the influences that parents, coaches, and peers had on their sport development and performance achievement. Consistent with previous research on this topic (Côté et al., 2007; Barreiros et al., 2013; Keegan et al., 2014; Atkins et al., 2015), this study suggests that parents, coaches and peers played an important role throughout volleyball players' development and affected players' experiences in sport as well as their expertise achievement.

The tangible support provided by parents of both highly skilled and less skilled players during the early years of development was perceived to facilitate their overall development. Here, parents provided the best conditions possible for their children's sport involvement, particularly the opportunity to engage in sport and all resources required for practice (i.e., financial support, equipment, transportation to and from training sessions and competitions, flexibility in rearranging family schedules). This type of support is considered a precondition for a sustainable and committed early sport involvement, building therefore a solid foundation for a prolonged engagement in sport and talent development (Côté et al., 2007; Lauer et al., 2010a; Harwood et al., 2012; Fraser-Thomas et al., 2013; Knight, 2016). While tangible support was to be expected for all players, parents' level of involvement and parenting styles revealed interesting differences between highly skilled and less skilled players. Highly skilled players emphasized the importance of parents' moderate level of involvement, explaining that they were present in important competitive moments for the player (example: official competitions, finals), but not present in trainings or informal competitive events (less significant for players). Highly skilled players also mentioned the importance of the autonomy provided by parents throughout development. Here, they explained that parents did not meddle in their decisions when it comes to the choice of a club or the negotiation of their contract. On the contrary, less skilled players reported an excessive parental involvement in their sport experience, represented by parents being always present in everything (formal or informal competitive moments, training sessions, etc.). They acknowledged this excessive involvement was a negative influence for their sport development and performance. Research examining parents' level of involvement in sport have shown that when parents adopt a moderate level of involvement in sport combined with autonomy-supportive behaviors (continuing attempts to encourage and allow their children to take responsibility for their own achievement performance and behavior), athletes tend to have positive experiences in sport such as higher levels of intrinsic motivation (Fraser-Thomas et al., 2008a,b; Lauer et al., 2010b; Barreiros et al., 2013; Preston and Fraser-Thomas, 2018). On the other hand, an excessive parental involvement is linked to athletes' negative sport experiences such as pressure, stress, negative emotional outcomes and burnout (Fraser-Thomas et al., 2008a,b; Fraser-Thomas and Côté, 2009; Harwood and Knight, 2009a,b). While these findings shed light on important insights regarding the positive and negative parental behaviors that influence volleyball players talent development, more research is needed to examine the specific behaviors that facilitate healthy sport experiences in highly skilled and less skilled players.

Coach behaviors also had a significant impact throughout players' development. Both highly skilled and less skilled players identified caring as an important characteristic of their coaches in the early years of development. This finding is corroborated by past research describing coaches' role in the early years as being kind, cheerful and caring, providing a playful introduction to sport (Côté, 2002; Côté et al., 2007; Fraser-Thomas et al., 2008b; Jones, 2009). The entrance in organized sport is a stressful moment for a child, so this motivational and cheerful environment offered by the coach may provide positive initial experiences captivating therefore children for a prolonged engagement in sport. Coach recognition of player's value was also a positive influence perceived by all players. This recognition was demonstrated in coaches' belief of players' potential to play volleyball, combined with a positive style of communication (Rollnick et al., 2020) and the opportunities provided for players to exhibit and develop their skills. This finding corroborates the results found by Fraser-Thomas and Côté (2009) showing that coaches' belief positively influenced adolescent swimmers' experiences in sport. Despite the importance of such findings, this body of research has been deeply overlooked in sport sciences. Therefore, additional research would benefit further understanding concerning the relationship between coach's recognition of player's value (or coach's belief) and athletes' outcomes in sport.

Of particular interest is the differences found between highly skilled and less skilled players concerning specific coach behaviors. Highly skilled players recognized that a rigorous and perceived demanding training environment combined with a very structured training plan and one-on-one instruction during adolescence were beneficial for their expertise development. These behaviors, and specially coaches' demand, were represented in the way they want the things to be done (exercises, the technical actions, the tactical positions, the understanding of the game, the overall commitment). Highly skilled players characterized their coaches as very committed in providing high level training practices, providing attention to technical and tactical development and pushing them to the limit so as they were able to achieve a high level of performance whenever possible. Highly skilled players interpreted this type of coach behaviors as beneficial for their development and they never considered it as excessive, negative or prejudicial for them. These findings are consistent with past studies showing that demanding coaching, individualized instruction and goal setting are important requisites to enhance athlete development and expertise achievement during the specializing years (Côté et al., 2007; Fraser-Thomas et al., 2008a,b; Cushion et al., 2012; Barreiros et al., 2013).

Finally, the findings of this study also highlight the important role of peers (within and outside sport) throughout athlete development (Côté et al., 2007; Keegan et al., 2010; Barreiros et al., 2013; Bruner et al., 2013). Specifically, both highly skilled and less skilled players recognized that friends were not only one of the main reasons to start playing volleyball, but also an important source of support for continued engagement and staying motivated for practicing sport. These findings are aligned with previous research clearly indicating affiliation with others and the development of positive social relations as major motives underlying children's interest in sport involvement (Weiss and Stuntz, 2004; Fraser-Thomas et al., 2008a; Fraser-Thomas and Côté, 2009). Nevertheless, clear differences relating to specific peers or teammates behaviors were found between highly skilled and less skilled players. Highly skilled players perceived teammates' criticisms and positive push (during practice) as an important factor for enhancing their motivation, team cohesion and friendship. These behaviors were perceived as positive rather than negative for the player and they believed this training environment contributed to potentiate their development and the achievement of high levels of performance. Within the study of peer interactions in sport (Keegan et al., 2009, 2010, 2014), researchers have suggested that a wide range of behaviors such as competitive behaviors, evaluative communication and social relationships may create an appropriate motivational atmosphere that strongly contributes to consolidate and strengthened friendship and team cohesion, enhancing athletes' motivation and facilitating therefore the athletes' healthy development in sport. Thus, the way athletes perceive such behaviors is of utmost importance to know how to deal with them. Furthermore, highly skilled players also described the importance of having friends outside of sport because they understand their demanding sporting life and agenda, while simultaneously act like an escape from all the pressure of volleyball during the later stages of their development. Consistent with literature (Côté et al., 2007; Fitzgerald et al., 2012; Bruner et al., 2013; Atkins et al., 2015), friends are an appropriate source of support during the later stages of development by fulfilling athletes' motivational and emotional needs. Notwithstanding, further studies examining peer support in sport should explore the athletes' and peers' backgrounds and contexts since this may have a great influence in the manner peers understand the sport involvement and its requirements.

Despite the important findings of this study, there are some limitations that should be addressed. Although widely used in literature, reliable and valid, retrospective methodologies only reflect interpretation of records and participants' reports/perceptions of their previous sport experiences, which need to be triangulated with other data (Sosniak, 2006). Future studies, though, may benefit from further exploring the use of qualitative research methods (such as focus groups, participant observation, action research, ethnographic studies) so as to provide a more consistent and enhanced understanding regarding the role of parents, coaches, and peers throughout players' development and expertise achievement. The use of such qualitative research methods could be specifically important to explore an integrated understanding of the inter-relationships that may exist between them and their influences on players' development. Here, longitudinal mixed methods approaches may also contribute to better understand these issues since may combine intervention, observation and practical reflection with multiple stakeholders (parents, coaches, athletes, other parents, peers) providing a more ecological and integrated perspective of these social influences throughout time. These studies could offer a more concrete understanding about the changes that the influence of parents, coaches, and peers may have throughout time according to the athletes' developmental stage. Future studies may also profit from the triangulation of different perspectives of distinct social agents rather than solely the athletes' perception (Pankhurst et al., 2012). Thus, examining the perception of parents, coaches and peers (within and outside sport) may provide a better, global, and solid picture of social influences in sport. In line with this perspective, further investigations should therefore explore in more detail what specific behaviors, attitudes and influences parents, coaches and peers exert that positively or negatively influence the development of an athlete and their expertise enhancement (e.g., supportive behaviors, how autonomy was provided by parents, types of leadership behaviors by coaches, types of behaviors provided by peers during training sessions and competitions that may interfere in the motivational climate, etc.). Here, more sport-specific studies are necessary to understand if the behavioral patterns showed by parents, coaches and peers change according to the idiosyncrasies of each sport as well as its cultural context. Finally, it is also important that future studies take into consideration the socioeconomic background of the social agents under study since this factor could interfere in the way they interpret sport and the interrelationships within this context.

## Data Availability Statement

The original contributions presented in the study are included in the article/supplementary material, further inquiries can be directed to the corresponding author.

## Ethics Statement

The studies involving human participants were reviewed and approved by Ethics Committee of Faculty of Sport of University of Porto. The patients/participants provided their written informed consent to participate in this study. Written informed consent was obtained from the individual(s) for the publication of any potentially identifiable images or data included in this article.

## Author Contributions

All authors listed have made a substantial, direct and intellectual contribution to the work, and approved it for publication.

## Conflict of Interest

The authors declare that the research was conducted in the absence of any commercial or financial relationships that could be construed as a potential conflict of interest.
